# A Comparative View of the Principal Theories, Which Have Prevailed in Chemistry; &c.

**Published:** 1799-04

**Authors:** 

**Affiliations:** Sen. of Vienna


### *A Comparative View of the Principal Theories, which have prevailed in Chemistry; &c.* By Dr. Frank, *Sen. of Vienna.*

[*Continued from No.* I. *page* 63—70.]

*Heat* and *Light** are, according to modern experiments, the effects of two different simple substances. They act either separately or in combination; in the latter case the result is called fire. Both are originally fluid bodies and provided by nature with the highest degree of expansibility. It is this property which renders them capable of diminishing or suppressing the power of attraction in all other bodies, and transforming them from a solid into a vaporific or elastic state, while they enter with these bodies, into chemical combinations, according to the greater or less affinity, or elective attraction subsisting between them.

*Air* and *Water* are not simple bodies, and therefore do not deserve the name of *elements*, which was given them by *Aristotle*; because both are compounded of parts which can be chemically separated. The *acids* and metallic calces likewise do not belong to the list of elementary substances; such as *carbon*, *sulphur*, *phosphorus* and the *metals*.

The air of the atmosphere is a compound of two elastic fluids. One of these is not only totally unfit for respiration, but likewise incapable of supporting the flame of combustible bodies. Hence it is called mephitic, suffocative air, or more properly, according to the modern French nomenclature, *Azote (gas azotique)*, or by the older Chemists, *phlogisticated air*. This irrespirable species of air constitutes about three-fourths of the whole atmosphere, with very little or no variation, in the most opposite climates; it is an element reduced to a gaseous form by the access of caloric, and we meet with it, as a constituent part of all animal and many vegetable bodies.

The second constituent element in the composition of the atmosphere, which forms about the fourth part of it, is that which renders it fit for respiration,

---

* In the doctrine of Light, the author of this essay follows the opinion of Professor Hermbstaedt, of Berlin, and not that of Lavoiser, who considers Heat and Light *modifications of the same substance.*

reſpiration, as well as combuſtion. This elaſtic fluid is a very remarkable agent in modern Chemiſtry, and its preſence is diſcoverable in the greater number of the phenomena which preſent themſelves to the inquirer. Its baſis is entirely different from that of azote, and it is found in all organized and many of the mineral bodies: and on account of its property of forming acids, it is now called the *acidifying principle*, or *oxygen*, in an abſtract ſenſe; but when it is exhibited to the ſenſes in a gaſeous ſtate, it is then termed *oxygenous gas*.

Beſides theſe, there are two other elementary ſubſtances, which deſerve particular attention in the New Syſtem of Chemiſtry—the *carbon*, or the baſe of charcoal, and the *hydrogen*, or the baſe of inflammable air. Both are conſtituent parts of all organized bodies: the former is the *pure charcoal*, but the latter cannot, independent of its combination with other ſubſtances, be exhibited to the ſenſes in any other but an elaſtic form, after having united with caloric; whence it has received the name of *hydrogenous gas*, or inflammable air.

Combuſtion can take place only in oxygenous gas, and depends upon a chemical decompoſition of this elaſtic fluid. All bodies of the animal and vegetable kingdoms, ſulphur, phoſphorus and the different metals, effect this decompoſition of oxygen in a high temperature, while they unite with the baſis of it in a gaſeous ſtate, and diſengage the reſpective matters of heat and light, from which this baſis derives its elaſticity; ſo that accordingly as theſe ſubſtances enter into new combinations, the reſult of ſuch unions is either light or heat.

In the combuſtion of organized bodies we find, that oxygen is alternately decompoſed by carbon and hydrogen, which always exiſt in them as conſtituent parts. The latter, *hydrogen*, by its union with *oxygen*, produces water, but which, in the moment of its origin, is moſt commonly again decompoſed into its original conſtituents by the co-operation of the carbon, and deprived of its oxygen, which, *after having united with it, and formed the carbonic acid (fixed air * aërial acid)*, eſcapes in a gaſeous form.

The

* Although it appears to have been unconnected with the deſign of Dr. Frank, to take notice in this Essay of every individual to whom the science of Chemistry is indebted for its rapid progress; yet we cannot, without injustice, omit to mention in this place, the radically important discoveries of Dr. Black of Edinburgh, respecting one of the most interesting airs, now called *carbonic acid*, as well as the sensible and latent properties of *caloric*. These may justly be considered as the introduction to Dr. Priestley's discoveries, which, together with those of *Bergman* and *Scheele*, in Sweden, have unquestionably laid the foundation of all the recent improvements in chemistry. It

The hydrogen thus disengaged, enters into a new combination; this becomes again decomposed, and the alternate combinations and decomposition in which light or heat are evolved, in this manner continue, until all the constituent parts of the combustible body have undergone this process of dissolution; and nothing but the earthly particles, combined with some insoluble and incombustible salts, remain behind.

These phenomena, during the combustion of animal and vegetable substances, however, take place only during the free access of atmospherical air; if this be secluded during the process, the result is very different.—As soon as the powerful agency of heat destroys the combination subsisting between the constituent parts, the hydrogen of the substance under experiment, uniting with oxygen, forms water; which is partly decomposed by the disengaged carbon, partly changed into vapour, together with the carbonic acid and the gaseous hydrogen generated in this process. In the mean time there arises in most vegetable substances, as well as in animal fat, a *new combination of the carbon and hydrogen*, which combination produces an acid by means of the oxygen present. If this latter be almost consumed, a new phenomenon occurs, and instead of an acid, the result is an *oil*, or a combination of *carbon*, and *hydrogen*, with an inconsiderable quantity of oxygen. In animal as well as in many vegetable bodies, on account of the *azote* and *hydrogen* they contain, *ammonia*, or vegetable alkali, is generated instead of an acid;—in other respects, the phenomena are similar to those already stated, with this only difference subsisting between vegetable and animal substances, that the former leave behind carbon deprived of hydrogen and oxygen, together with a few earthy and saline particles; while the latter exhibit a residuum of

---

It would be unpardonable to withhold from these illustrious characters that share of merit and praise to which they possess claims scarcely inferior to those of the French chemists. We principally allude to the original discoveries made by Dr. Black, relative to the well-established properties of *magnesia*, *fixed air*, and *latent heat*. His useful labours, during a long series of years, as a luminous and celebrated lecturer, have been crowned with a degree of success almost unexampled in the annals of science. Perhaps no professor of chemistry ever enjoyed so much honour and satisfaction as has fallen to the lot of Dr. Black: he has educated a greater number of respectable pupils than any public teacher now living; inspired the learned as well as the dilettanti of this country with a just taste for the prosecution of chemical studies; and, upon the whole, he has been essentially instrumental in improving that science, by his excellent method of analytically and experimentally demonstrating chemical truths; a method no less elegant than convincing.—On account of ill health, and the rapid advancement of age, he has lately been obliged to resign his academical chair to his former adjunct, the present ingenious professor HOPE; who, from his long residence in Paris, and the habits of intimacy he has cultivated with the most eminent French chemists, is equally zealous and able to establish the antiphlogistic system, in the Edinburgh school, upon a solid and permanent basis. W.

of carbon, combined with the phosphat of lime, or a calx saturated with the phosphoric acid.

In the combustion of *sulphur* and *phosphorus*, we observe phenomena very different from those which present themselves in combustible organized bodies. Sulphur and phosphorus merely attract oxygen; they form in this combination peculiar acids, and thereby disengage the respective elements of heat and light which appear in the form of flame. The acid generated from sulphur and oxygen, if the former be completely saturated with the latter, is termed *sulphuric acid (acide sulphurique)*; but, if a portion of sulphur still remains unsaturated, it is then called the *sulphurous acid (acid sulphureux)* so that the different termination of the last syllable is always used to indicate the degree of saturation in the acid. In a similar manner is obtained from phosphorus, either the *phosphoric* or *phosphorous acid.*

The *calcination* of *metals* is likewise a process of *combustion.* These substances combine with *oxygen,* lose their lustre and cohesion, and in this form are termed *metallic oxyds (oxides metalliques).* Some of these, however, such as *arsenic, molybdæna,* (black lead and *tungsten,* are capable of uniting with a larger portion of oxygen, and are convertible into *true acids.*

Hence the products of combustion are *water, acids,* and *metallic oxyds.* Their absolute weight is uniformly greater than that of the substance from which they are obtained; because the oxygen, which unites with them, is a ponderous substance. A clear proof of this assertion is the correspondence of the additional weight in the oxyds, with that of the oxygen gas absorbed by the metals.

But the process of combustion is not the only one, in which oxygen enters into combination with other substances:—the process of respiration; the spontaneous decomposition of metals; electricity; the contact of bodies with those substances to which oxygen is either loosely attached, or in which it exists in an abundant proportion, for instance, the acids; the process of fermentation, &c. all these afford opportunities for the oxygen to form combinations.

In the respiration of animals, the oxygen gas is separated by the lungs; during this admirable process in the animal economy, it partly incorporates with the blood, and partly, with the base of carbon it meets with, forms the carbonic acid, which, together with the portion of azote inspired from the atmosphere, escapes in the subsequent expiration. As, however, the caloric of separated oxygen gas is more than sufficient for reducing the carbonic acid to an elastic form, the surplus of caloric is necessarily communicated to the animal body, which consequently receives its constant supply of heat, from the more or less regular evolution of oxygen gas.

Those

Thofe mineral productions which contain fulphur, as a conftituent part of the ores of metals, are fubject to fpontaneous decompofition. The mechanical part of this fimple procefs is as follows: metallic ores are placed in ftrata, over one another; water is poured over them, and thus they are expofed, for fome length of time, to the influence of the atmofphere, till they acquire a vitriolic tafte. The confequent refult is, in part, to be afcribed to the decompofition of the water, by the action of the metal, which deprives the former of its oxygen, and difengages the fecond conftituent part of the water, hydrogen, which efcapes in a gafeous form; and it partly depends on this circumftance, that the fulphur attracts as much oxygen from the atmofphere, as is neceffary for its change into an acid. The product muft be a metal, faturated with the fulphuric acid; for inftance, the fulphat of iron.

The *acidification* of *azote* is accomplifhed by the influence of *electric matter*. This takes place either by the fpontaneous effect of atmofpheric air, and its natural electricity, on animal and vegetable fubftances which contain azote in abundance, as is the cafe in nitre-works; or by means of an artificial procefs, in which electric fparks are paffed through a mixture of azote and oxygen. In both cafes the *acid* of *nitre* arifes, which according to the degree of its faturation with oxygen, is either called gafeous oxyd of nitrogen, *nitrous gas, nitrous acid (gas nitreux, acide nitreux)*, or if fuperfaturated with oxygen, *nitric acid* (*acide nitrique*).

By treating or mixing fubftances with acids, thofe which have a greater chemical affinity or elective attraction to oxygen, than the bafis of the former, viz. the acids, have to the latter, they likewife become oxydated. Such is the cafe when metals are diffolved in acids. The acid employed is, in this operation, regularly decompofed; the oxygen attaches itfelf to the metal, and reduces it to the ftate which it has affumed by combuftion, or in other words, it becomes an oxyd. At this period only, the metal is in a proper condition of being received by the remaining undecompofed part of the acid, and a complete folution of it is accomplifhed. On thefe occafions, a gafeous fubftance is always evolved, but of a different nature, in different inftances. If the nitric acid be ufed as a menftruum, the confequent expulfion will be that of nitrous gas; becaufe one part of this acid is deprived of fo much oxygen, that the bafis of nitre alone—a combination of azote with a fmall portion of oxygen, remains behind, which, by the free caloric, then prefent, acquires the elaftic form.

In making fimilar experiments with the *concentrated* fulphuric acid, fulphurous gas efcapes; on the contrary, hydrogenous gas, when it has been

*diluted*

*diluted* with water; and this for the following reaſon: in the former caſe, one part of the ſulphuric acid parts with much of its oxygen, hence ſulphurous acid muſt be the product; this, however, in the ſecond caſe, meets in the water a body, from which, on account of its greater power of attraction, it derives as much oxygen as it had been deprived of by the metal; conſequently, as the water is compoſed of oxygen and hydrogen, the latter muſt naturally become free, and diſengage itſelf in a gaſeous form.

*Farina*, *mucilage*, *gum*, *ſugar*, are educts, rather than products, which are ſeparated from vegetable bodies, either by the operations of nature, or by ſimple chemical proceſſes. All theſe are combinations of *carbon* and *hydrogen*, in different proportions, with a portion of *oxygen*. They readily aſſume the nature of an acid, particularly when they meet with an opportunity of attracting a large portion of oxygen; on which account, all ſubſtances, capable of being converted into acids, are generally termed *acidifiable baſes*. This combination with oxygen, may be accompliſhed, either by employing an acid which contains this element but looſely combined, as for inſtance, the nitric acid, or by means of fermentation.

The former of theſe operations is very ſimple, and conſiſts merely in immerſing the acidifiable ſubſtance in nitric acid, till ſuch time as red fumes are no longer expelled. The nitric acid is thus completely decompoſed; it is deprived of the greateſt part of its oxygen, and compelled to eſcape in the elaſtic form of nitrous gas. The ſubſtance ſubjected to ſolution, on the contrary, abſorbs its oxygen, and is transformed into an acid.

All *vegetable acids* have ſimilar baſes, namely, *carbon* and *hydrogen*. The whole and only difference ſubſiſting between them, depends upon the *relative* and *different* quantity of the oxygen by which they are acidified. The *acetous acid* is the moſt perfect among them, and all others are, in part, changed into it, by a more perfect ſaturation with oxygen. Some acids of the vegetable kingdom, however, likewiſe contain azote and phoſphorus; but whether theſe two are eſſential ingredients of them, has not yet been aſcertained.

If vegetable ſubſtances containing farinacious, mucilaginous, and ſaccharine ingredients, are expoſed to a temperature above 10° of Reaumur, or $54\frac{1}{2}$° of Fahrenheit, the equilibrium ſubſiſting between their conſtituent parts is thus deſtroyed. Theſe are now ſet at liberty to enter into new combinations. A great part of the free carbon unites with oxygen, and eſcapes as carbonic acid; the hydrogen, however, in combination with another part of carbon and oxygen, produces alkohol, or an inflammable ſpirit. This is called, in chemical language, the firſt, or vinous fermentation. Alkohol, being an acidiacidifiable body, then combines not only with the portion of oxygen ſtill exiſting in the mixture, but it likewiſe attracts ſo much of it from the atmoſphere, that, after being changed into an acid, it appears in the form of vinegar. Theſe are the limits of the ſecond ſtage of fermentation. Laſtly, in the third ſtage, hydrogen is diſengaged; and, if azote be one of the conſtituents in the ſubſtance under experiment, the product is *ammonia*, which derives its origin from the baſes of *hydrogen* and *azote*. Theſe ſucceſſive productions take place till the whole vegetable body is reduced to its earthy baſis, and the more ſolid inſoluble ſalts. Every organic ſubſtance, however, does not exhibit theſe phenomena, during its fermentation. Several plants afford no alkohol, while animal ſubſtances yield neither this nor an acid. Thoſe of the former kind immediately paſs over into the acetous, and the latter, into the fetid, or putrefacted fermentation.

In reviewing the different methods, by which oxygen forms combinations, it is evident that it is *excluſively this elementary ſubſtance* that *forms the acids*, and transforms or changes all ſuch ſubſtances into acids, as are capable of receiving it in conſiderable quantity. Although the baſes of ſome of the acids, for inſtance the *muriatic*, *fluoric* (*fluor-ſpathic*), and *coracic*, are hitherto *unknown*, yet reaſon and analogy intitle us to conclude, with a high degree of probability, that they, like all others, have individual baſes which from the oxygen have acquired their acid nature, but which cannot be exhibited in a ſeparate ſtate, becauſe their affinity to oxygen is ſo great, that they cannot be attached to any other ſubſtance. The capacity of the muriatic acid to abſorb an additional portion of this ſubſtance, and to form what is called ſuperſatured or oxygenated (dephlogiſticated) muriatic acid, affords an obvious confirmation of this conjecture.

It is further obſervable, that *metals* aſſume the form of calces or oxydes, only when they enter into combination with oxygen, In that combination alone they belong to the claſs of *compound* bodies; whereas in their *metallic* ſtate we are unable to decompoſe them, and therefore muſt conſider them as *ſimple* bodies. Laſtly, it follows that *water* conſiſts of *hydrogen* and *oxygen*, and as ſuch cannot be called a ſimple body.

But theſe propoſitions are not merely ſynthetically true; they can be verified alſo by a ſtrict analyſis. As oxygen muſt be neceſſarily ſubject to the laws of chemical affinity, all its combinations muſt be liable to be diſſolved, and the reſpective bodies with which they were formed, to be reduced to their priſtine ſtate; as ſoon as, under the requiſite conditions, another body operates upon them, whoſe elective attraction for oxygen is greater than that which attaches or fixes it to their baſis. In moſt inſtances, pure carbon is a ſubſtance of of this nature. In a *high* temperature, by the degree of which the elective attractions in general are very much influenced, carbon deprives the acids of their oxygen, appears in the form of carbonic acid, and completely diſengages their baſes, for inſtance, the ſulphur, from the ſulphuric acid; the phoſphorous from the phoſphoric acid, &c. It *decompoſes water*, if it be made to paſs over it in the form of vapour conducted through red-hot tubes; combines with the oxygen of it, and thus diſengages the hydrogen. It *reduces* the metallic oxydes when at the degree of fuſion, and reſtores to them their metallic properties. Some of theſe oxydes, however, thoſe of the *precious metals*, and of *mercury*, do not ſtand in need of carbon to diſengage their oxygen. They readily part with it, in a temperature ſomewhat higher than that in which they have abſorbed it; becauſe in this the affinity of oxygen to caloric is greater than to the baſis of the metal; it eſcapes in the form of oxygenous gas, and may be eaſily intercepted, incloſed in glaſs or other air-proof veſſels, and exhibited to the ſenſes.

In an analogous manner, as bodies in their combination with oxygen acquire additional weight, they, in the contrary caſe, when diſoxygenated, or deprived of their acidity, loſe a part of their abſolute weight. This loſs, however, can be as eaſily accounted for as the former increaſe; it originates in the eſcape of the oxygen, and its relative amount is, in every inſtance, proportionate to the weight of the latter.

Thoſe readers who have not yet acquired a familiar knowledge of the antiphlogiſtic ſyſtem, will be enabled from this ſhort ſketch to form, at leaſt, a ſuperficial eſtimate of its merits. The firſt and original works, which appeared on this ſubject, are the two following: 1. "*Opuſcules phyſiques et chymiques, par Lavoiſier*:" à Paris; Tom. i. ii. 8vo. 1774; and, 2. "*Traité elementaire de Chimie, par Lavoiſier*:" à Paris; 8vo. 1789.

---

Having now briefly delineated the elements of the modern French ſyſtem of Chemiſtry, it remains to ſtate, conciſely, the various modifications which this ſyſtem has lately undergone in Germany, or rather, to ſketch an outline of the later ſyſtems to which it has given birth.

Profeſſor Voigt, of Jena, ſome years ago, publiſhed a new chemical theory, which remarkably deviated from the former phlogiſtic, as well as the preſent antiphlogiſtic ſyſtem*.

The

---

* *Verſuch einer nuen Theorie, &c.* i. e. *An attempt towards eſtabliſhing a new theory of fire, of combuſtion, of the different gaſes, of reſpiration, fermentation, electricity, of the meteors, of light and magnetiſm:* by Profeſſor Voigt, Jena, 8vo. 1794.

The learned Profeſſor adopts *two* kinds of phlogiſtic, or *inflammable ſubſtances*, viz. *male* and *female*; which, however, are not cognizable by our ſcales or inſtruments of menſuration. The former exiſts in all *combuſtible* bodies of the animal and vegetable kingdoms, in *ſulphur*, *phoſphorus*, and the *metals;* it forms with *water* the *male inflammable gas* (inflammable air; hydrogen gas): the latter, when combined with water, exhibits the *female inflammable gas* (pure vital air; oxygen gas), and, as ſuch, conſtitutes the fourth part of atmoſpheric air.

The remaining part of the atmoſphere (phlogiſtic air; azote) is an *elementary air*, which cannot be condenſed into a fluid vapour; it is neither fit for reſpiration nor combuſtion, but is capable of diſſolving a variety of ſubſtances.

*Light* is an extremely ſubtle matter, every where extended, which however can ſenſibly affect the eye only, when its parts ſuſtain a violent degree of vibration.

The *acids* are formed by a peculiar *acidifying baſis*, which exiſts in all bodies affording acids, but which is *concealed* in them by means of the male baſe of inflammability; as, for example, in phoſphorus, in ſulphur, in the carbon, &c.

In a ſimilar manner, a peculiar *alkaline baſis* produces the *alkaline ſalts*, which, according to its different modifications, are at one time *fixed*, and at another *volatile*.

*Water* is an *elementary* ſubſtance, which, indeed, can be *ſeparated*, by means of combuſtion, from the male and female baſes of inflammability, and again combined with theſe baſes, according to particular circumſtances, but it cannot be *decompoſed* into its conſtituent parts.

*Carbon* is formed of the *aërial acid*, and the *male baſis of inflammability*. The former is a ſubſtance that is indebted to the acidifying element for its origin, which *cannot be decompoſed*, and which, in its gaſeous ſtate, is combined with a portion of water.

The different *earths* we meet with in nature, are modifications of one and the ſame ſimple ſubſtance, the *baſis of earth*.

If an inflammable body come in contact with the female inflammable gas, and if by any ſpecies of vibration, for inſtance by friction, the male inflammable baſe of the former be kindled, there takes place a *reciprocal action* between the two inflammable baſes; they unite; and if this copulation (or, as Profeſſor Voigt expreſſes himſelf, this grinding and milling operation of their nuptials be *calmly* accompliſhed), *heat* or *actively combined inflammable matter*

*matter*, is the refult. And if the reciprocal concuffion of the two principles be attended with *violence*, the *matter of heat* is at the fame time difengaged, and *fire* is confequently produced.

The inflammable bafe, thus actively combined, at length paffes over into a *calm* ftate, and is diffolved by the *fimple* air of the atmofphere. The product of this folution is called inflammable air (phlogifticated air; azote.)

All bodies which are deprived of their male bafis of inflammability, acquire an addition of weight, and new properties. The acids, for inftance thofe of phofporus, of fulphur, become free; the metals are reduced to the form of calces. Their male bafe of inflammability combines with the female, emanated from the female inflammable gas; thus the water of the latter is difengaged, which attaches itfelf to the body fubject to combuftion, and muft, therefore, neceffarily increafe its abfolute weight.

As foon, however, as fuch bodies, for inftance calciform metals, the phofphoric acid, &c. meet with an opportunity of depriving another fubftance, fuch as the carbon, of its male inflammable bafe, according to the fixed laws of affinity, they again return to their former ftate, the phofphoric to the phofphorus, the metallic calces to bright metals, &c. The water they have abforded, combines, in this cafe, with the carbonic acid, difengaged from the carbon, and efcapes in the form of gas. As, however, the water was the only caufe of their increafed weight, the male principle of inflammability, which they exchanged in return, does not manifeft any fenfible weight, it is obvious that fuch bodies muft be reduced to that weight which they poffeffed previous to their combuftion.

In an analogous manner, the author adopts the fexual fyftem in the doctrines of electricity and magnetifm: he calls the two oppofite magnetic poles, as well as the *plus* and *minus* in electricity, by the fanciful terms of *male* and *female*.

---

Lefs artfully conftructed, more confiftent, and incomparably better adapted to explain the phenomena of nature, is the fyftem lately propofed by the celebrated chemift of Pruffia, Dr. Richter, whofe works are well known to the learned of France, as well as of Britain.

(To be continued in our next number.)

